# Multi-Order Brain Functional Connectivity Network-Based Machine Learning Method for Recognition of Delayed Neurocognitive Recovery in Older Adults Undergoing Non-cardiac Surgery

**DOI:** 10.3389/fnins.2021.707944

**Published:** 2021-09-16

**Authors:** Guoqing Wu, Zhaoshun Jiang, Yuxi Cai, Xixue Zhang, Yating Lv, Shihong Li, Guangwu Lin, Zhijun Bao, Songbin Liu, Weidong Gu

**Affiliations:** ^1^School of Information Science and Technology, Fudan University, Shanghai, China; ^2^Department of Anesthesiology, Huadong Hospital, Fudan University, Shanghai, China; ^3^Institutes of Psychological Sciences, Hangzhou Normal University, Hangzhou, China; ^4^Department of Radiology, Huadong Hospital, Fudan University, Shanghai, China; ^5^Department of Geriatric Medicine, Huadong Hospital, Fudan University, Shanghai, China

**Keywords:** delayed neurocognitive recovery, resting-state functional magnetic resonance imaging, functional connectivity, machine learning, sparse representation

## Abstract

**Objectives:** Delayed neurocognitive recovery (DNR) seriously affects the post-operative recovery of elderly surgical patients, but there is still a lack of effective methods to recognize high-risk patients with DNR. This study proposed a machine learning method based on a multi-order brain functional connectivity (FC) network to recognize DNR.

**Method:** Seventy-four patients who completed assessments were included in this study, in which 16/74 (21.6%) had DNR following surgery. Based on resting-state functional magnetic resonance imaging (rs-fMRI), we first constructed low-order FC networks of 90 brain regions by calculating the correlation of brain region signal changing in the time dimension. Then, we established high-order FC networks by calculating correlations among each pair of brain regions. Afterward, we built sparse representation-based machine learning model to recognize DNR on the extracted multi-order FC network features. Finally, an independent testing was conducted to validate the established recognition model.

**Results:** Three hundred ninety features of FC networks were finally extracted to identify DNR. After performing the *independent-sample T test* between these features and the categories, 15 features showed statistical differences (*P* < 0.05) and 3 features had significant statistical differences (*P* < 0.01). By comparing DNR and non-DNR patients’ brain region connection matrices, it is found that there are more connections among brain regions in DNR patients than in non-DNR patients. For the machine learning recognition model based on multi-feature combination, the area under the receiver operating characteristic curve (AUC), accuracy, sensitivity, and specificity of the classifier reached 95.61, 92.00, 66.67, and 100.00%, respectively.

**Conclusion:** This study not only reveals the significance of preoperative rs-fMRI in recognizing post-operative DNR in elderly patients but also establishes a promising machine learning method to recognize DNR.

## Introduction

Clinically, the neurocognitive impairment including memory, information processing, and execution identified from 7 to 30 days post-operatively is defined as delayed neurocognitive recovery (DNR) (Berger et al., 2015; Evered et al., 2018). With the improvement of medical order and the development of aging society, more and more elderly patients are prone to suffering DNR (Evered et al., 2017) after undergoing surgery under anesthesia. The increase in DNR not only causes heavy medical and social burden but also brings troubles to families. Hence, it is crucial to understand the underlying pathological mechanisms of DNR for the prevention and treatment of it.

Recent studies have shown that preoperative cognitive function decline may be an important risk factor for cognitive impairment following surgery and anesthesia (Hogue et al., 2006), and some preoperative brain functional and structural features related to DNR have been identified. Zhang et al. (2019) found that there were differences of regional homogeneity in the right hippocampus between DNR patients and non-DNR patients. Jiang et al. (2020) found that the preoperative higher amplitude of low-frequency fluctuation (ALFF) in the bilateral middle cingulate cortex (MCC) and lower functional connectivity (FC) between the bilateral MCC and left calcarine were independently associated with the occurrence of DNR. Although these studies have identified possible preoperative neuroimaging risk factors for DNR, the correlation between different factors was not further considered in the research process, and a stable and reliable DNR patient recognition model based on those factors has not been established.

Resting-state functional MRI (rs-fMRI) has provided a useful tool to analyze the brain response and cognitive impairment by describing both the localized neural activity and the connection characteristics of the entire brain network (Rubinov and Sporns, 2010; Lei et al., 2020a). In most rs-fMRI-based brain function studies, brain regions are usually considered as vertices, and their functional interactions calculated by averaging correlations of blood oxygenation order–dependent (BOLD) signals across the whole scanning session are considered edges to build a brain FC network. However, since neural synchronization shifts very quickly to meet cognitive demands, this calculation method of functional interaction makes it difficult for the brain network to effectively describe the neural behavior in the temporal dimension. Recently, dynamic FC based on spatial–temporal joint analysis of BOLD time signal sequences has become a powerful and promising framework in brain network research (Chang and Glover, 2010; Kiviniemi et al., 2011; Hutchison et al., 2013; Calhoun et al., 2014). These dynamic FC methods utilize a sliding window to divide the entire time series of the BOLD signal into numerous segments of sub-series in advance and then establish a series of temporal FC networks based on each segment of the signal. Since the adjacent networks should share a similar topological pattern and connection strength, some recognized changes can be utilized as discriminative information to identify abnormal brain networks (Chen et al., 2017; Zhao et al., 2018).

The higher-order FC features that regard fixed-position brain regions as vertices and the interaction between brain regions as edges are playing an increasingly important role in brain network–based diagnostic research. Chen et al. (2016) combine the extracted low-order and high-order FC networks to discriminate between early mild cognitive impairment (eMCI) and healthy controls and achieve a promising classification accuracy of 88.14%. Zhao et al. (2018) propose to use a multi-level, high-order FC network representation for autism spectrum disorder (ASD) diagnosis. Experimental results show that the integration of both low-order and first-level high-order FC networks achieves the best ASD diagnostic accuracy (81%). Moreover, they further find that the high-order FC features can provide complementary information to the low-order FC features in the ASD diagnosis. Lei et al. (2020b) build a machine learning model based on the combination of high-order and low-order features to recognize global cognitive impairment in moyamoya disease (MMD). Experimental results demonstrate that high-order features play a more important role than low-order features in the classification model.

In the present study, based on the hypothesis that there are differences in preoperative brain FC between DNR and non-DNR patients, we first establish a set of multi-order brain FC network feature extraction methods to quantify both the connections between the activities of two brain regions and the connections between the activities of brain region groups. Then, we investigate the discriminative features between the patients with and without DNR by simultaneously analyzing the correlation between features and categories and the redundancy between features. Finally, we build a complete set of machine learning classification methods to identify DNR based on the mined discriminative features.

## Materials and Methods

### Participants

A total of 308 patients collected from September 2017 to February 2019 at the Huadong Hospital Affiliated to Fudan University were enrolled in this study. Included and excluded criteria (Jiang et al., 2020) are detailed as follows. The included criteria were the following: (1) patients scheduled to undergo non-cardiac surgery with an expected duration of more than 2 h, under general anesthesia; (2) age ≥ 60; and (3) American Society of Anesthesiologists classification I–III. The excluded criteria were the following: (1) education duration <6 years, (2) Mini-Mental State Examination (MMSE) score prior to surgery <24 points, (3) pre-existing mental and/or psychiatric disease, (4) Parkinson’s disease, (5) history of cardiac and/or central nervous system vascular disease, (6) history of cardiac and cranial surgeries, (7) taking sedatives or antidepressants during the nearest year, (8) alcohol or drug abuse, (9) severe hepatic or renal dysfunction, (10) vision and audition impairment or language troubles impeding communication, (11) situations unsuitable for an MRI scan, and (12) unwillingness to complete repeat neuropsychological tests. We use the Z-score method to determine whether DNR occurs after surgery. Specifically, we first calculate the difference between the baseline score and the post-operative score of a certain neuropsychological test. Then, we calculate the difference between the two scores of the healthy control group before and after the test. Finally, we subtract the difference of the control group from the difference of the patient group and calculate the Z-score by dividing the result by the standard deviation of the difference between the two scores of the control group. When the Z score of two or more tests was more than 1.96, the patient was judged to have DNR. Anesthesia protocols and neurocognitive assessment have also been published in our previous study (Jiang et al., 2020); for more details, please refer to it. After the evaluation of the included and excluded criteria, the final data of 74 cases, including 16 cases of DNR and 58 cases of non-DNR, were used for experimental model building and testing. This was a nested case–control study approved by the Ethics Committee of Huadong Hospital Affiliated to Fudan University with the approval number of 20170020.

### Image Acquisition and Pre-processing

All MRI scan were performed at least 1 day prior to surgery on a 3.0 T MRI scanner (Skyra, Siemens, Munich, Germany). The imaging parameters of rs-fMRI data included 33 axial slices, slices of thickness = 4 mm with a 0-mm gap, *TR* = 3,000 ms, *TE* = 30 ms, voxel size = 3.4 × 3.4 × 4 mm^3^, and flip angle = 90°. In this scan, 120 volumes were obtained. Data preprocessing procedures were performed with Statistical Parametric Mapping (SPM12, The Wellcome Trust Centre for Human Neuroimaging, London, United Kingdom) and RESTplus version 1.22 (Institutes of Psychological Sciences, Hangzhou Normal University, Hangzhou, China) in MATLAB version R2019b (MathWorks, Inc., Natick, MA, United States) (Ren et al., 2016).

The first five volumes of each case were discarded to reduce the potential noise interference of the imaging instrument. The remaining volumes were subjected to slice-time correction, realignment, and spatial smoothing with a 6 × 6 × 6 mm^3^ Gaussian kernel in turn. After the linear trend of time course removal, temporal filtering (0.01–0.08 Hz) were performed to remove the effects of low-frequency drift and high-frequency noise. The cerebrospinal fluid and white matter signal were regressed out as nuisance covariates. Finally, the volumes were spatially normalized to the Automated Anatomical Labeling (AAL-90) template and further partitioned into 90 regions of interest (ROIs) (Tzourio-Mazoyer et al., 2002).

### Feature Extraction of Brain FC Network

**FIGURE 1:**
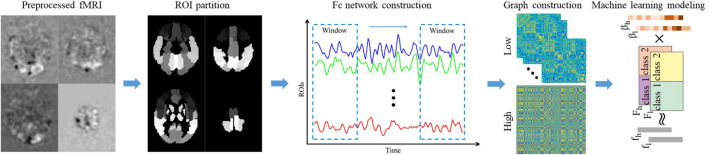
The overall framework of the proposed method. The pre-processed fMRI is registered to the ALL brain template and divided into 90 ROI regions in advance. Then calculate the average gray value of each ROI regions to get the time series of each ROI region. Convert the time series of all ROI regions into low-order FC network diagrams through sliding window operation and correlation calculations. Perform clustering and correlation analysis of the low-order FC network to obtain the high-order FC network. Finally, extract the low/high-order FC network graph features, and establish machine learning model to recognize DNR.

In order to construct a brain FC network, rs-fMRI images need to be converted into brain activity signals. As shown in Figure 1, by averaging the voxels of each brain region of each volume, we can get a time series, which reflects the change in the activity of each brain region. Then, we divide the time series into several sub-series in the time dimension through the sliding window. Denoted by t, w, and s the length of the original signal, sliding window size, and sliding step size, respectively, there will be K = ((t−w)/s)1 sub-series after the sliding window operation. Denoted by ${\text{x}}_{i}^{j}\left(n\right)$ the *j*-th sub-series in the *i*-th ROI of case *n* and denoted by X^*j*^$\left(n\right)=\left\{{\text{x}}_{1}^{j}\left(n\right),{\text{x}}_{2}^{j}\left(n\right),\ldots {\text{x}}_{90}^{j}\left(n\right)\right\}$ the *j*-th sub-series in total 90 brain regions of case *n*, the brain network FC matrix A^*j*^(*n*) corresponding to the *j*-th sub-series of case *n* can be obtained by calculating the Pearson’s correlation strength between two different ROIs (Salvador et al., 2005):


$${\text{a}}_{p,q}^{j}\left(n\right)=\text{c}orr\left({\text{x}}_{p}^{j}\left(n\right),{\text{x}}_{q}^{j}\left(n\right)\right)$$

where *p* and *q* represent two ROIs. Regarding each ROI as a node and the strength of the correlation coefficient between regions as an edge, then a K dynamic brain FC network can be obtained for each case. In our experiment, we set the sliding window size and sliding step size to 90 and 1, respectively.

To describe the characteristics of the brain FC network more comprehensively, we further construct the high-order dynamic network by calculating the correlation between the elements of the low-order connection matrix. Specifically, denoted by $y_{p,q}\left(n\right)=\left\{a_{p,q}^{1}\left(n\right)\right\},\left\{a_{p,q}^{2}\left(n\right)\right\},\ldots ,\left\{a_{p,q}^{K}\left(n\right)\right\}$ and $y_{u,v}\left(n\right)=\left\{a_{u,v}^{1}\left(n\right)\right\},\left\{a_{u,v}^{2}\left(n\right)\right\},\ldots ,\left\{a_{u,v}^{K}\left(n\right)\right\}$ the element sets of the *p*−*q*-th and the *u*−*v*-th positions in the K low-order FC networks, the high-order correlation between the *p*−*q*-th element and the *u*−*v*-th element can be calculated as


$${\text{h}}_{p,q,u,v}\left(n\right)=\text{c}orr\left({\text{y}}_{p,q}\left(n\right),{\text{y}}_{u,v}\left(n\right)\right)$$

This high-order dynamic network not only describes the relevance of the activities of multiple groups of brain intervals but also integrates the entire time dimension information to achieve a higher temporal and spatial resolution of the description of brain FCs. In fact, since the range of *p*, *q*, *u*, *v* is all 1–90, the high-order brain network of one case has 90^4^ elements, which may not only lead to time-consuming computation but also contain some redundant information. Thus, we use an element time series clustering method to convert the element–order correlation (90 × 90 vs. 90 × 90) into group correlation (300 vs. 300), where 300 is the number of clusters. When obtaining the low/high-order FC networks, we establish weighted undirected graphs for feature extraction. Note that for the K low-order networks, we perform feature extraction on the averaged network. Based on the undirected graphs, we extract local clustering coefficients to quantify the brain function network. It describes the degree to which a node in the graph and its neighbor nodes gather to form a clique (complete graph) and can be calculated as (Strogatz, 2001)


$${\text{c}}_{i}=\frac{2\sum_{j\in N_{i}}{\left({\text{w}}_{ij}\right)}^{1/3}}{\left|{\text{N}}_{i}\right|\left(\left|{\text{N}}_{i}\right|-1\right)}$$

where *N_i_* denotes the number of neighbors of node *i* and *w*_*ij*_ is the weight between nodes *i* and *j*.

### Machine Learning Modeling

Ninety low-order and 300 high-order FC network features were finally extracted for each subject. These features contain some redundant information, which will not only increase the computational complexity of the subsequent classification model but also increase the risk of model overfitting (Wu et al., 2019a). Therefore, before establishing the classification model, we first perform feature dimensionality reduction. In recent years, feature selection based on sparse representation has achieved encouraging performances in many radiomics studies (Wu et al., 2018, 2019b). Compared with traditional statistical estimation-based feature selection methods, such as independent-sample t-test, sparse representation feature selection can consider both the correlation between features and labels and the redundancy between features. Therefore, we utilize sparse representation to select a few of discriminative features for subsequent classification. The model can be formulated as


$$\hat{\varphi }=\underset{\varphi }{\text{argmin}}{||l-F\varphi ||}_{2}^{2}+\eta {||\varphi ||}_{0}$$

where *l* denotes the sample label set; *F* = [*f*_1_;;*f*_2_;;*f*_*J*_] denotes the sample feature set, *J* is the number of samples; and ^η^ is a regularization parameter and is set to 0.1 in our experiments. The absolute value of each element in the representation coefficient $\hat{\varphi }$ indicates the importance of the corresponding feature. Once the elements of $\hat{\varphi }$ are calculated, we rank the features according to the values of $\hat{\varphi }$ in descending order in advance. Then, we use apply a cross-validation-based sequential forward selection strategy to select the optimal feature subset (Zhu et al., 2016). Specifically, first, we take the top five features as the initial feature subset and evaluate the cross-validation classification effect of the training data set based on the current feature subset. Then, through the loop, we put the 6th to 100th features into the feature subset in turn and evaluate the model classification effect after each feature subset update. When the loop ends, we can find the optimal model classification result, and the feature subset corresponding to the result is the optimal feature subset.

We build a classification model on the selected features to predict DNR. In fact, our classification problem has two challenges. On the one hand, the number of experimental samples are relatively small (*n* < 100). On the other hand, the ratio of positive and negative samples is quite different (16 vs. 58). The sparse representation classifier (Wright et al., 2009) uses a non-parametric training classification method, which reduces the model complexity and the risk of model overfitting under small samples. The model can be formulated as


$$\hat{\beta }=\underset{\beta }{\text{argmin}}{||\bar{f}-\bar{F}\beta ||}_{2}^{2}+\gamma {||\beta ||}_{0}$$

where $\bar{f}$ denotes the testing sample feature after feature selection, $\bar{F}=\text{[}{\bar{F}}_{1},{\bar{F}}_{2}{\bar{F}}_{C}]$ denotes the feature set of the training sample after feature selection, and *C* is the total number of sample categories. γ is sparse representation of control parameters, which is set to 0.05 in this study. When the sparse representation coefficient $\hat{\beta }$ is obtained, calculate the residual:


$$r_{c}\left(\bar{f}\right)=\bar{f}-\bar{F}\delta_{c}\left(\hat{\beta }\right),c\left[1,2\ldots C\right]$$

where δ_*c*_(⋅) is used to select the coefficient corresponding to the c-th feature. The final testing sample category is finally determined by $ID\left(\bar{f}\right)={argmin}_{c}r_{c}\left(\bar{f}\right)$. It can be seen from the working principle of the sparse representation classifier that it is a non-parametric training classifier, so there is no model training stage. Based on the idea of K proximity classification, in the testing phase, it directly compares the similarity between the test sample and the training sample between different categories to determine the category of the test sample. In particular, the similarity between samples here is measured by calculating the characteristic Euclidean distance between samples. In addition, in the classification process, thanks to the sparse constraints, the classifier can effectively grab the most essential features of the sample and suppress redundant features, thereby reducing the adverse effects of the difference in the number of positive and negative samples.

In the model validation stage, we first divide our data into a training set and an independent testing set according to a ratio of 2:1. Then, we perform feature selection and 10-fold cross-validation on the training set. Finally, we train the model on the training set and test the model on the independent testing set. Metrics including the area under the receiver operating characteristic curve (AUC), accuracy (ACC), sensitivity (SEN), and specificity (SPE) (Yu et al., 2020; Zheng et al., 2020) are calculated to evaluate the performance of our method.

## Results

Seventy-four patients who completed both preoperative and post-operative MRI scans and all neuropsychological tests were used for the final experimental analysis. Among these 74 patients, 16 cases were diagnosed as DNR and 58 cases were diagnosed as non-DNR. According to statistics, the education order of DNR patients was significantly lower than that of non-DNR patients, while in terms of age, gender, height, weight, and obesity, there was no difference between the two groups of patients. Table 1 reports a detailed comparison of the statistical results of the two groups of patients.

**TABLE 1:** Baseline characteristics.

Variables	DNR (16)	Non-DNR (58)	*P*
Age (years)	63.5 (62.0, 67.0)	64.0 (61.0, 68.3)	0.598
Sex (male/female)	12/4	29/29	0.075
Education (year)	6 (6, 9)	9 (9, 12)	0.002
Height (m)	1.68 (0.08)	1.65 (0.08)	0.185
Weight (kg)	59.0 (50.0, 70.5)	60.0 (54.8, 70.0)	0.324
BMI > 24, n	3 (18.8)	19 (32.8)	0.437
Smoking, n	7 (43.8)	15 (25.9)	0.281
Surgical history, *n*	6 (37.5)	28 (48.3)	0.444

*In the statistical results of the age, education, and weight, the data in brackets give the minimum and maximum values of the variable.*

**FIGURE 2:**
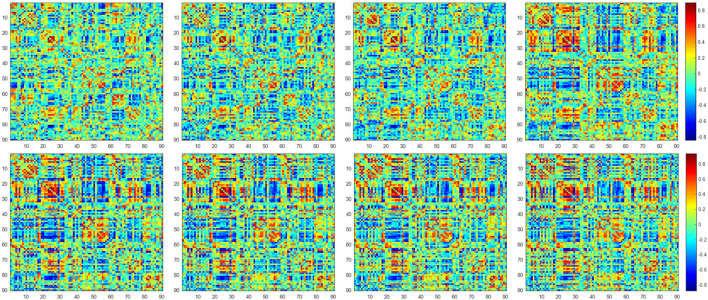
The constructed low-order FC networks. Each sub-figure corresponds to a different dynamic sliding window. Each element in the matrix is the correlation between two brain regions through the pairwise Pearson’s correlation analysis. Element with light color indicates positive correlation, while the dark color shows a competitive or anti-correlation relationship between regions.

**FIGURE 3:**
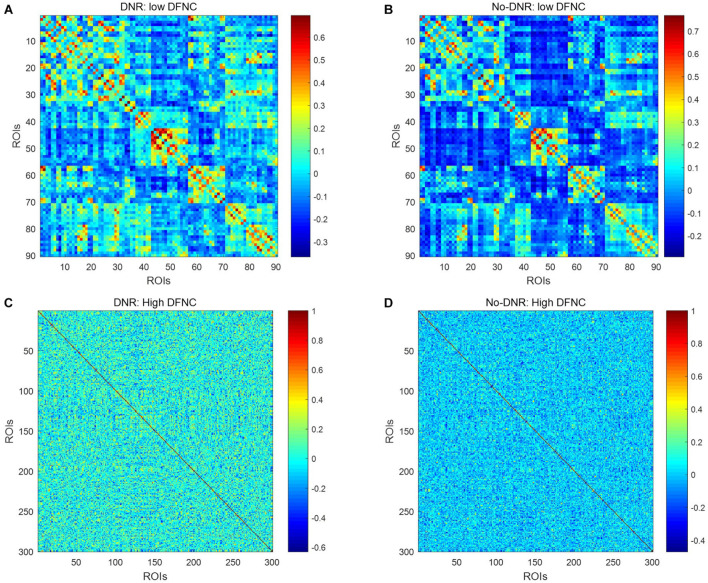
Averaged low\high-order FC networks for all patients. Panels **(A–D)** are the low-order FC network of DNR, the low-order FC network of No-DNR, the high-order FC network of DNR, and the high-order FC network of No-DNR, respectively. For panels **(A,B)**, each element in the matrix is the correlation between two brain regions. For panels **(C,D)**, each element in the matrix is the correlation between two clusters. Element with light color indicates positive correlation, while the dark color shows a competitive or anti-correlation.

Figure 2 shows the dynamic FC network obtained after sliding window processing on the DNR patient time series signal, where each sub-figure corresponds to a set of sliding window data. It can be seen that there are certain differences in the correlation between the activities of different brain regions in the FC network constructed from the same signal with different time windows. This implies that the dynamic FC network based on the sliding window can effectively extract the time window information of the brain activity. In addition, as shown in Figures 3A,B, we average the FC network connection matrix of all patients and all sliding windows in the DNR and non-DNR groups, respectively, to visually compare the brain network activity of the two groups. The brighter the region in the figure, the stronger the connection between the corresponding two brain regions. In addition, it is obvious that there are more connections among ROIs in the DNR group. The high-order FC network is used to measure the correlation between elements in the low-order FC network. However, the matrix generated by directly calculating the correlation between all elements in the low-order FC network not only contains a lot of redundant information but also increases the calculation amount of the subsequent analysis model. Therefore, based on the clustering method, we first cluster the elements in the low-order FC network according to the time series in all sliding windows of the elements and then calculate the correlation between the various types to build the final high-order FC network. The averaged high-order FC networks of the DNR and no-DNR patients are compared in Figures 3C,D. Consistent with the comparison results in Figures 3A,B, the DNR group exhibits more clusters with positive correlations. The latent and discriminative features in low/high FC networks are quantified in the following part.

**FIGURE 4:**
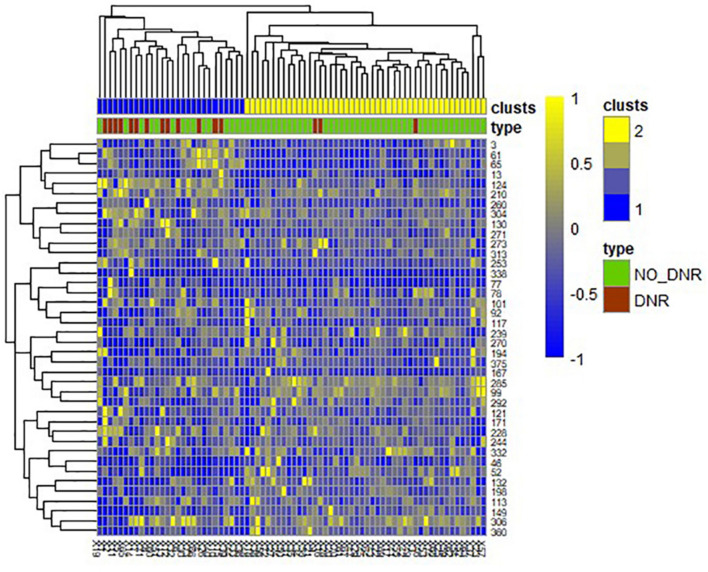
Unsupervised clustering heat map of the selected 40 features. Rows represent features, columns represent samples. In the first two rows, blue and yellow represent the results of automatic clustering of samples, and red and green represent the true categories of samples.

**FIGURE 5:**
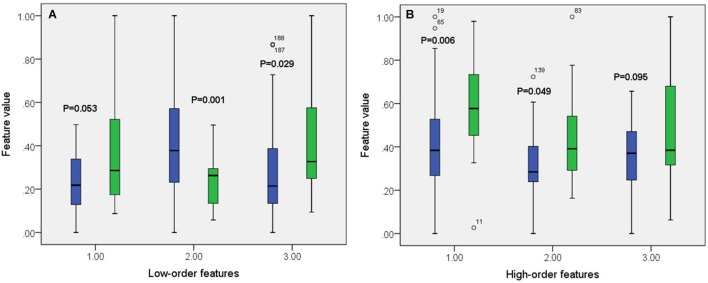
Boxplot of some features from both low-order **(A)** and high-order **(B)** networks. The green color corresponds to DNR group while the blue color corresponds to no-DNR group.

**FIGURE 6:**
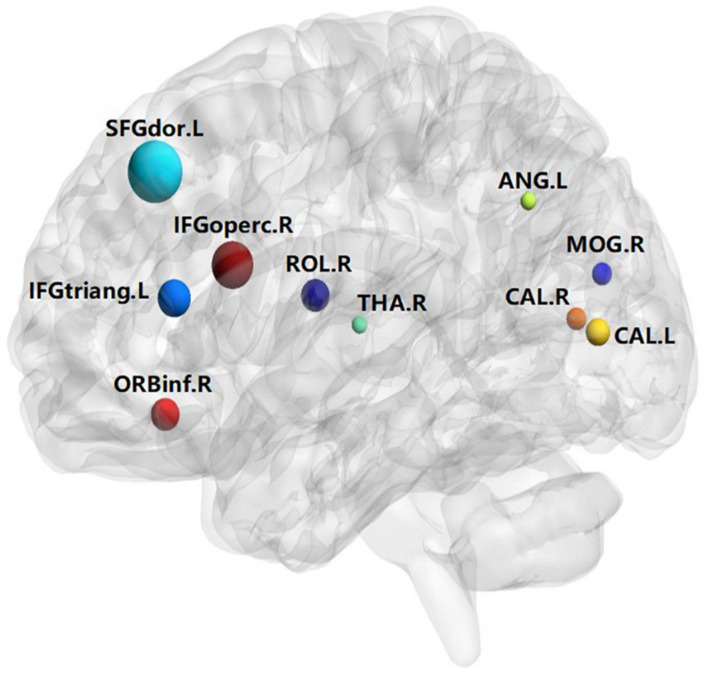
Visualized ROIs selected from low-order FC networks.

**FIGURE 7:**
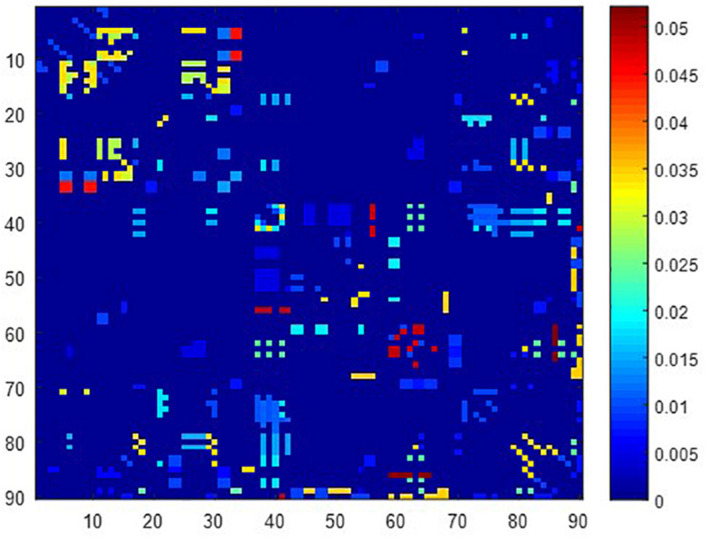
Visualized ROIs selected from high-order FC networks, the brighter the color is, the more important a cluster is.

A total of 390 local clustering coefficient features were finally extracted for each patient, where 90 features were extracted from the low-order FC network and 300 features were extracted from the high-order FC network. The low-order FC network features correspond to the 90 brain regions one to one. *Independent-sample t-test* results between these features and the categories show that 15 features are statistical differences in DNR identification (*P* < 0.05) and 3 features are significant statistical differences (*P* < 0.01). After sparse representation-based feature selection (the detailed feature selection process is provided in section “Machine Learning Modeling”), 40 features are selected for the final machine learning modeling, of which 10 features are from the low-order FC network and 30 features are from the high-order FC network. The results of the unsupervised clustering heat map (Liu et al., 2019) of the 40 features are shown in Figure 4. In the first two rows, blue and yellow represent the results of automatic clustering of samples and red and green represent the true categories of samples. After automatic clustering, 56 out of 74 cases obtain the correct category label, which shows the effectiveness of these features. In addition, we choose three features that had the minimum *p*-value from both low-order and high-order sets to depict their boxplots in Figure 5. For the selected 10 low-order FC network features, we select the 10 brain regions corresponding to them and visualized them in Figure 6. Figure 7 shows the importance visualization of the 30 high-order FC networks obtained by feature selection. These 30 high-order FC networks actually correspond to 30 low-order FC network clusters. In the feature selection step, the feature selection model in section “Machine Learning Modeling” calculates the importance values of these high-order FC networks, which are the absolute values of $\hat{\varphi }$. We assign these 30 importance values to the corresponding 30 low-order FC network clusters to obtain the result in Figure 7. Therefore, the brighter area corresponds to the cluster with a larger importance value obtained by feature selection; that is, the cluster is more important.

**FIGURE 8:**
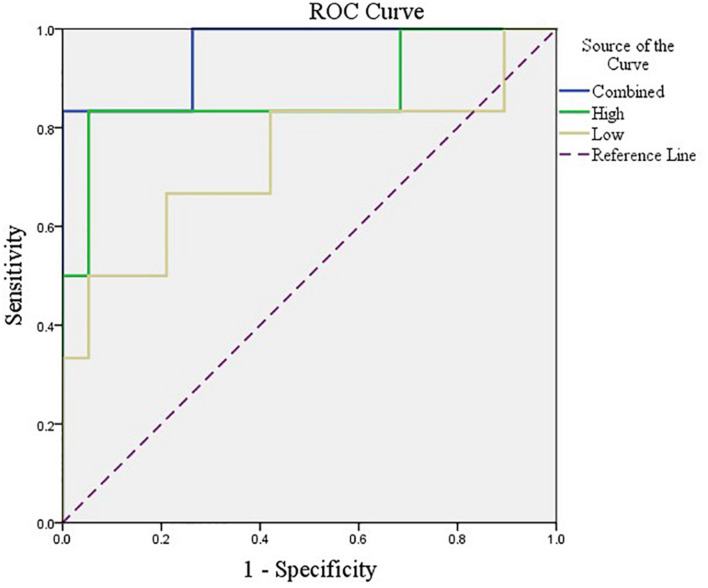
ROC curves corresponding to the three methods.

After feature selection, the sparse representation-based classification model was trained on 49 training data in advance, and then the trained model was tested on the rest of 25 testing data. The final DNR recognition results are reported in Table 2, “Low-order feature,” “High-order feature,” and “Combined,” respectively, indicate that the features for model training are only low-order features, only high-order features, and a combination of the two features. The combined method achieves the best performance with AUC, ACC, SEN, and SPE of 0.9561, 0.9200, 0.6667, and 1.000%, respectively. Figure 8 shows the ROC curve comparison of the three methods.

**TABLE 2:** Comparison of recognition results on the independent test set.

Method	AUC	ACC	SEN	SPE
Low-order feature	0.7368	0.7600	0.5000	0.8421
High-order feature	0.8684	0.8800	0.5000	1.0000
Combined	0.9561	0.9200	0.6667	1.0000

## Discussion

The results of this study show that the preoperative brain FC network features can be used to predict post-operative DNR by establishing an effective machine learning model. The key findings include the following: (1) The correlation between the activities of different brain regions in DNR patients before surgery is stronger than that in non-DNR patients. (2) The activity of 10 brain areas, namely, Frontal_Sup_L, Frontal_Inf_Oper_R, Frontal_Inf_Tri_L, Frontal_Inf_Orb_R, Rolandic_Oper_R, Calcarine_L, Calcarine_R, Occipital_Mid_R, Angular_L, and Thalamus_R, is highly correlated with whether the patient suffers from DNR after surgery. (3) Based on the multi-order brain FC network features, the established machine learning DNR recognition model in this study has achieved promising results and may provide some guidance for clinical anesthesia surgery for the elderly.

Recently, an increasing number of clinical studies have reported that whole-brain-based FC analysis plays a primary role in predicting post-operative cognitive impairment. Jiang et al. (2020) found that preoperative lower FC between the bilateral MCC and left calcarine was more prone to have post-operative DNR. The results of literature (Zhuang et al., 2019) showed that patients with cognitive impairment had abnormal regional activities and FC of calcarine. Literature (Moon and Jeong, 2017) further proves that lower spontaneous activities in the calcarine were independently associated with cognitive impairment. In addition, Zheng et al. (2017) confirmed that patients with amnestic cognitive impairment, severe cognitive deficits, and major depression disorder all exhibited abnormal FC.

Although these studies have examined the preoperative alterations in the brain FC structure with post-operative cognitive impairment and found some consistent findings, most of these studies have the following shortcomings. Firstly, as far as the research subjects are concerned, most of them only pay attention to the relationship between certain brain region activities and post-operative cognitive impairment, without considering the entire brain network activity. Since most brain activities are jointly participated by multiple brain regions, considering only one or a few functional connections of brain regions may ignore some key information that affects the judgment. Secondly, as far as the research process is concerned, they only use some simple statistical estimation methods to explore the factors that may be related to post-operative DNR, and the synergistic effects of multiple features are rarely considered. Finally, most of these methods only reveal some features related to post-operative DNR but do not establish a complete post-operative DNR prediction model, which may provide guidance for the treatment of elderly post-operative patients.

Compared with these existing studies, our study has the following advantages. First, we explored the relationship between the whole preoperative brain network activity and post-operative cognitive impairment by establishing a feature extraction method based on the combination of low-order and high-order brain networks. It can be seen from the results in Table 2 that high-order FC network features are the key to the good results in the proposed method. A low-order network feature is proficient in measuring the correlation between the activities of any two brain regions in a time-varying way, but it cannot describe the correlation between multiple brain regions. Some existing studies (Lei et al., 2020b) have shown that the correlation of changes in multiple groups of brain regions plays a more important role in FC network-based brain disease diagnosis. Therefore, we build a clustering-based high-order FC network to quantify the correlation between different pairs of brain regions, so as to further explore deeper interaction relationships. Secondly, we utilize sparse representation-based feature selection method rather than traditional statistical estimation methods, such as independent-sample *T*-test, to mine high-resolution features related to post-operative DNR. This feature selection method considers both the correlation between features and tags and the redundancy between features during feature selection, so that it can effectively select a feature subset that best expresses the sample category. Finally, more importantly, we have built a complete DNR prediction model and obtained encouraging prediction results.

**FIGURE 9:**
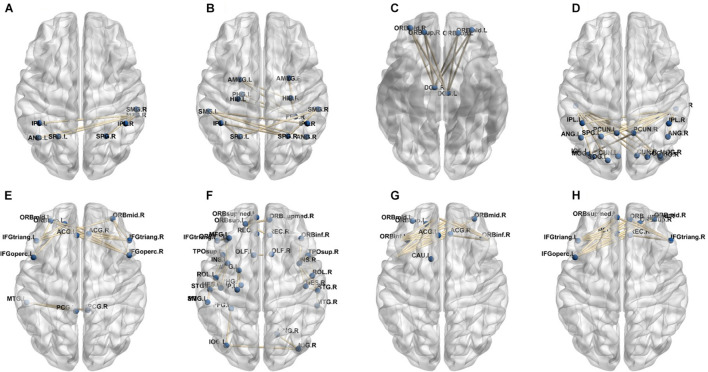
Clusters of low-order FC network (1–8). **(A–H)** First to eighth clusters.

**FIGURE 10:**
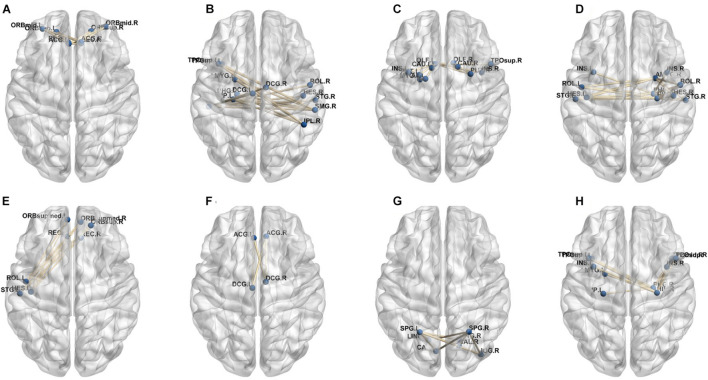
Clusters of low-order FC network (9–16). **(A–H)** Ninth to sixteenth clusters.

**FIGURE 11:**
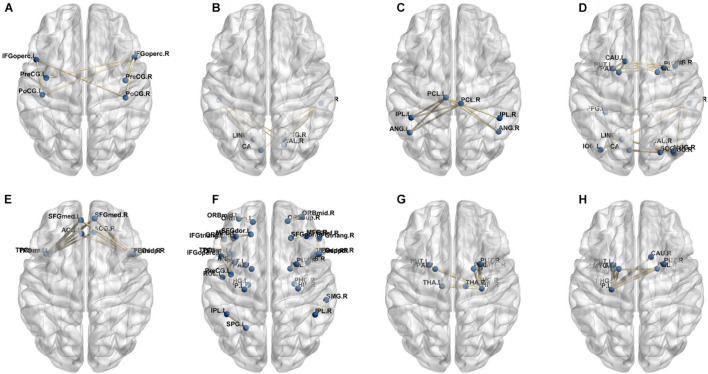
Clusters of low-order FC network (17–24). **(A–H)** Seventeenth to twenty-fourth clusters.

**FIGURE 12:**
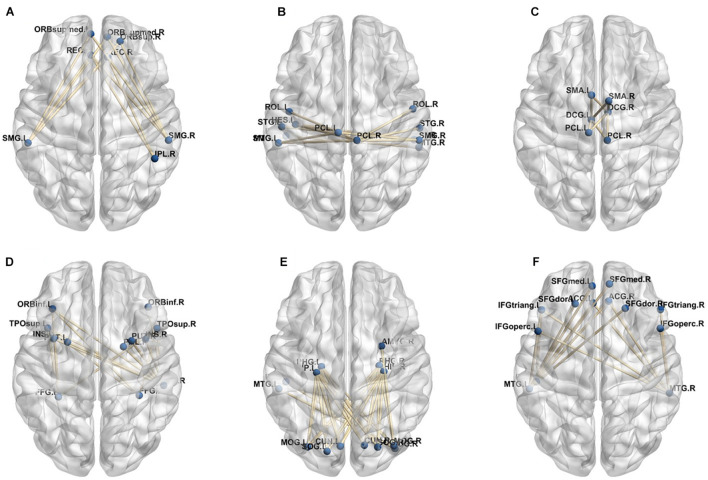
Clusters of low-order FC network (25–30). **(A–F)** Twenty-fifth to thirtieth cluster.

In our deep learning-based classification model, 30 high-order FC networks are selected for the final DNR recognition. Furthermore, these 30 networks correspond to 30 clusters of low-order FC networks. According to the reviewer’s suggestions, we first visualized the 30 clusters and then discussed these network connections and brain regions. Figures 9–12 show the 30 clusters, where each brain network subgraph corresponds to a cluster of low-order FC network. From these figures, we can see that the activity of the symmetrical brain region has a high correlation. For example, in Figures 9C,D, 10A,F, 11B,E, 12B,F, many symmetrical brain connections are grouped into the same cluster. We further count the number of occurrences of each brain region in all of these 30 clusters, among which the top 10 brain regions with the most occurrences are listed as follows in turn: Insula_R, Hippocampus_L, ParaHippocampal_L, Insula_L, Temporal_Pole_Sup_L, Temporal_Pole_Sup_R, Parietal_Inf_R, Frontal_Sup_Orb_L, and Frontal_Inf_Tri_L. According to previous reports, most of these brain regions are related to patients’ cognition, learning, and memory. For example, the human insula has been found to contribute to its crucial role in goal-directed behaviors and emotional regulation, through rapid processing of attentional, cognitive, interoceptive, emotional, and autonomic signals (Craig, 2009). The effective connectivity analyses studied in Diersch et al. (2021) revealed that the age-related learning deficits were linked to an increase in hippocampal excitability. Chen et al. (2013) also found that the reduction in hippocampal volume before surgery is one of the risk factors for post-operative cognitive dysfunction (POCD) in elderly patients. The experimental results in Velia et al. (2013) showed that the separation of cognitive and sensory nerves has plasticity in the human superior temporal gyrus cortex. As shown in Figure 9B, the second important cluster of our model, Fusiform, is directly connected to Hippocampus and ParaHippocampal. These connections have been proved to directly affect the improvement of individual memory, so we speculate that the features corresponding to these connections may play an important role in DNR prediction. For the explanation of some other high-order FC features, since there is relatively little research in this research area, and our machine learning model integrates all selected features for classification, it is difficult for us to directly interpret the relationship between the occurrence of DNR and a certain FC feature. This is also the main work we need to complete in the future.

There are three limitations in our study. First, the diagnosis results of all cases are based on the post-operative neuropsychological tests performed at 7–14 days after surgery. Due to the influence of other post-operative factors, such as pain and inflammatory activity, the diagnosis of post-operative cognitive impairment in this period of time may be too early. Although the patients enrolled in our experiment do not experience pain and other symptoms during neuropsychological testing, the possibility of these effects cannot be completely ruled out. Second, the amount of experimental data is limited. A sufficient amount of data is the guarantee for the reliability and stability of machine learning models. In our experiment, 74 cases of data were used for model training and testing. Although we conducted strict training and testing data division, the stability of the model results still needs to be verified on more data sets, especially multi-center data. Therefore, in future work, we will further collect a large amount of multi-center data to verify the proposed model. Third, we build a dynamic FC network by manually setting a fixed-scale sliding window instead of in an adaptive way. Since the signal itself has certain changing characteristics, setting an adaptive sliding window will more effectively reflect the essential characteristics of the signal. Therefore, some engineering improvements will be made in the future to address this problem.

## Data Availability Statement

The raw data supporting the conclusions of this article will be made available by the authors, without undue reservation.

## Ethics Statement

The studies involving human participants were reviewed and approved by the Chinese Clinical Trial Registry (Identification number: ChiCTR-DCD-15006096). The patients/participants provided their written informed consent to participate in this study.

## Author Contributions

All authors listed have made a substantial, direct and intellectual contribution to the work, and approved it for publication.

## Conflict of Interest

The authors declare that the research was conducted in the absence of any commercial or financial relationships that could be construed as a potential conflict of interest.

## Publisher’s Note

All claims expressed in this article are solely those of the authors and do not necessarily represent those of their affiliated organizations, or those of the publisher, the editors and the reviewers. Any product that may be evaluated in this article, or claim that may be made by its manufacturer, is not guaranteed or endorsed by the publisher.
